# How men influence young women taking PrEP: perspectives from young women, male partners, and male peers in Siaya county, Western Kenya

**DOI:** 10.1186/s12905-024-03044-9

**Published:** 2024-04-03

**Authors:** Kawango Agot, Jacob Onyango, Brian Perry, Nneka Molokwu, Jamilah Taylor, Duncan Ngoje, Amy Corneli

**Affiliations:** 1 Impact Research and Development Organization, Mito Jura Road, off Kisumu-Kakamega Highway, Kisumu, 9171 - 40141 Kenya; 2 Department of Population Health Sciences, Duke University, Durham, NC USA; 3 Duke Clinical Research Institute, Duke University School of Medicine, Durham, NC USA

**Keywords:** Young women, Male partner, PrEP, HIV, Sexual partners, Kenya

## Abstract

**Background:**

Daily oral pre-exposure prophylaxis (PrEP) is an effective HIV prevention option for those who are most vulnerable to HIV infection, especially young women (YW). Objection by or lack of support from male sexual partners has been shown to impact YW’s ability to take PrEP consistently. We explored the views of YW, and male partners and male peers of YW in Siaya County, Western Kenya, to illustrate how men influence, and can support, YW in using PrEP.

**Methods:**

We used Photovoice to capture the views of YW ages 18–24 who were currently or previously enrolled in the DREAMS program and with current or previous experience taking PrEP. We also captured the views of YW’s sexual partners and male peers. The YW completed eight photo assignments that focused on identifying factors influencing their PrEP use, and male participants completed four photo assignments focused on identifying ways men support or hinder YW’s PrEP use. Photographs were presented and discussed in same- and mixed-gender groups using the SHOWeD method. YW also participated in in-depth interviews. The analysis focused on identifying themes that described men’s influence on YW’s PrEP adherence and persistence.

**Results:**

Among YW, a restricting male influence on PrEP use emerged in the majority of photo assignments such that YW’s photographs and discussions revealed that men were more often viewed as barriers than supporters. YW perceived that they had little autonomy over their sexual lives and choice to use PrEP. YW’s PrEP use was perceived to be hindered by stigmatizing community narratives that influenced men’s support of PrEP use among women. Male participants suggested that men would support YW’s PrEP use if PrEP was better promoted in the community and if men were more knowledgeable about its benefits.

**Conclusions:**

A lack of support from male partners and peers and stigmatizing community narratives influence YW’s PrEP use. Community-based programs should include education about PrEP specifically for male partners and peers of YW to positively influence PrEP use among YW.

## Background

Adolescent girls and young women (AGYW) ages 15–24 years are disproportionately affected by the HIV pandemic and account for approximately one-third of all new HIV infections globally [1–3]. In sub-Saharan Africa, AGYW account for 25% of new HIV infections among adults and have three to five times the prevalence of adolescent boys and young men [4–6]. In 2018, AGYW accounted for 26% of all new HIV infections in Eastern and Southern Africa [2] and one-third (30%) of the 41,728 new HIV infections in Kenya [7].

Various studies have demonstrated that HIV risk in young women (YW) is driven by socio-cultural, behavioural, and biological vulnerability [3, 8]. Those most at risk for HIV infection are characteristically from socio-economically disadvantaged households in areas with a high HIV prevalence, have limited or no education, engage in transactional sex, and have a history of sexually transmitted infections and/or pregnancy [9–11]. Shannon and colleagues have argued that unequal gender power dynamics in relationships are associated with gender-based violence and men’s control over sexual decision-making in relationships, which can impact women’s negotiation skills and space [12]. The existence of certain harmful cultural practices that require women to observe sexual rituals, such as widow cleansing and inheritance, also place young widows at risk for HIV [8, 13, 14]. For YW, income inequality and a lack of income (or a lack of control over income) may contribute to transactional sex or early marriage [15, 16]. Those who are economically deprived may also engage in high-risk behaviours, such as early sexual debut and engagement in transactional sex (sex for gifts or favours rather than formal sex work) [17, 18], in most cases with older partners with a higher likelihood of having HIV due to higher-risk sexual behaviours [9–11]. The amplifying cycles of socio-cultural, behavioural, and economic vulnerability undermines women’s decision-making and autonomy with regard to their health [8, 19, 20].

One of the most effective HIV prevention interventions is pre-exposure prophylaxis (PrEP). In September 2015, the World Health Organization (WHO) recommended that PrEP be offered as an additional prevention choice for people at substantial risk for HIV infection [21]. However, despite multiple trials demonstrating the effectiveness of PrEP [22–26], the large-scale rollout of PrEP among women in sub-Saharan Africa remains a challenge [27, 28]. Studies in regions of Kenya with a high HIV burden have reported low PrEP uptake and low continuation rates among AGYW initiated on PrEP through routine maternal and child health/family planning clinics. In a study conducted in Kisumu County, Western Kenya, Oluoch et al. reported that of 357 AGYW enrolled for PrEP, only 4% initiated PrEP at their family planning visit [29]. In another study also conducted in Kisumu County, Mugwanya et al. [30] found that of 278 (22%) AGYW who initiated PrEP, only 114 (41%) went for at least one refill visit after initiation. Another implementation science study on the PrEP adherence cascade conducted in Western, Central, and Coastal Kenya by Were et al. [31] indicated that of 3,138 AGYW eligible for PrEP, a total of 2,900 (92%) initiated PrEP, but only 914 (32%) and 154 (6%) continued to be on PrEP at months 1 and 3, respectively.

Studies have shown that male partners often exert considerable influence on their female partners’ use of female-controlled or female-initiated HIV prevention methods, such as PrEP [32–35]. A number of studies have also documented the relationship dynamics between YW and their male partners [36–38]. Although some YW can maintain persistent use of PrEP without the support of their male partners [39], YW often experience limited support from their partners for using HIV prevention methods and must balance their need to protect themselves against HIV with fear of their partner’s reactions and possible violence [34, 40, 41]. A women’s desire to preserve their relationship and the trust of their partner may weigh more on their lives than their risk of acquiring HIV and prevention considerations [35, 42]. As part of a larger study to identify the multi-level factors influencing PrEP use among YW [43, 44], we used a participatory-research approach to engage young women as well as male partners and male peers of YW to illustrate, from their perspectives, specific ways in which men influence YW’s PrEP use and to identify practical ways in which men can support YW in taking PrEP.

## Methods

### Study design

Between March and September 2019, we used Photovoice to capture the views of YW, and male partners and male peers of YW on factors influencing YW’s use of PrEP. Photovoice is a community-based participatory research method where people identify, represent, and engage their community using photography and critical reflection [45, 46]. Participants use photographs and stories to highlight salient personal, shared, and structural issues with others. Through discussion of the photographs, participants are able to communicate specific aspects of an experience, identify with others’ experiences, and reveal the realities depicted in the photographs. We also conducted follow-up in-depth interviews (IDIs) with YW to further explore themes that emerged from the Photovoice activities.

### Participant recruitment and eligibility

YW were recruited through the Determined, Resilient, Empowered, AIDS-free, Mentored, Safe (DREAMS) program, a U.S. Government-supported program that aims to reduce the burden of HIV among AGYW in sub-Saharan Africa. At the time of study recruitment, the DREAMS program and this research were implemented by the same organization. Mentors engaged by the DREAMS program referred YW to the study. To be eligible, YW must have been 18–24 years of age at the time of PrEP initiation, currently or previously enrolled in the DREAMS program, and currently or previously taking PrEP (as determined by participant self-report and DREAMS program records). Men must have been 18 years or older and identified by YW in the DREAMS program or through community outreach activities as a peer or sexual partner of YW. Both YW and men were also asked to refer others in their social networks to the study. All participants must have been willing to share their photographs and stories with others in order to participate. During the consenting process, participants were informed they will be asked to describe their photographs/stories in same-gender groups and mixed-gender groups with male partners and peers, as well as have the group discussions audio-recorded.

### Data collection

All participants received inexpensive, digital cameras. They were trained on how to use the camera and on ethics-related information when taking photographs, such as obtaining a signed photo release form from any person who was identifiable in a photograph and not taking photographs of people under 18 years of age.

Over several months, participating YW and men were given photo assignments to identify factors in their community that influence YW’s adherence and persistence to PrEP. The YW’s eight photo assignments were to take pictures that represent: (1) things that influence a YW’s sexual health, (2) situations in which YW’s risk of HIV is low, (3) situations in which YW’s risk is high, (4) what helps YW to take PrEP daily, (5) what hinders YW to take PrEP daily, (6) reasons YW stop taking PrEP for a short period of time, (7) reasons YW stop taking PrEP for long periods of time, and (8) ways that YW at risk for HIV can be supported and motivated to take PrEP continuously over time.

The men’s four photo assignments focused on taking pictures that primarily showed how male peers and partners influence YW’s adherence and persistence to PrEP and included pictures that represent: (1) how men (who live with YW/do not live with YW) can help YW’s use of PrEP, (2) how men (who live with YW/do not live with YW) can hinder YW’s use of PrEP, (3) situations where women feel like they no longer need PrEP but remain at risk for HIV, and (4) things that male peers and partners can do to support and motivate YW at risk for HIV to continue taking PrEP over longer periods of time.

After each photo assignment, participants chose the photographs that they wanted to present during the group discussions, worked with study staff to write descriptions of their images, and first shared and discussed their photographs and descriptions during same-gender group discussions. Participants could also ask study staff to present their photograph anonymously during the group discussions if they were uncomfortable sharing their photograph/story with others. The SHOWeD method was used to encourage discussion around the socio-ecological drivers of the issues [45, 46]. In following this method, participants were asked to describe their photographs by answering the following questions: What is being Seen? What is really Happening? How does the situation relate to Our lives? Why does this situation Exist? What can we Do about it? The discussions were facilitated by trained tri-lingual (English, Kiswahili, and Dholuo) researchers. Participants were encouraged to consider factors beyond what was visually depicted within the frame of the photograph and to connect how these factors relate to a particular topic of interest (e.g., PrEP adherence).

Transcripts from the same-gender group discussions were rapidly analysed to identify key issues that emerged in the photographs and discussions (described in the next section). Next, we conducted mixed-gender group discussions between the YW and men to further discuss key issues. During these discussions, participants shared and discussed photographs that were selected from the original photo assignments on similar themes that both the YW and men identified. Once all the mixed-gender group discussions were completed, YW participated in IDIs to further discuss key themes that emerged during the discussions and reflect upon how these themes affected their personal lives.

### Data processing and analysis

All Photovoice discussions and IDIs were audio-recorded with participant permission and simultaneously transcribed and translated into English. We used a rapid analysis process [47] to review the same-gender group discussion transcripts, photographs/descriptions, and moderators’ notes of key findings from the discussions. Two senior members of the research team independently reviewed the data, created matrices to display concepts discussed in each of the same-gender group discussions, and identified emerging factors across the participant cohorts. The emerging factors then became the themes we explored during the mixed-gender group discussions.

We used applied thematic analysis [48] to formally analyse the same- and mixed-gender group discussions and the IDI narratives. Information from the rapid analysis of the same-gender group discussions were used to create an initial structural coding framework to categorize information in the same- and mixed-gender group discussion transcripts. The IDI transcripts were coded using a similar process but with a different a priori coding framework, which mapped to the interview guide. Inter-coder reliability was assessed on 20% of the transcripts to ensure different analysts interpreted the structural codebooks and applied codes in similar ways. Coding reports of the various a priori topics were generated and independently reviewed by two qualitative analysts to identify emergent content-based codes that described participants’ statements about and experiences within the broad categories. The analysts then met to discuss their codes/themes and develop a content-based coding framework to apply to the structural coding reports. Analysts pretested the content codebook by independently coding a portion of each coding report corresponding to the a priori topics and then meeting to discuss their code application. Any coding discrepancies were discussed, and the codebooks were revised to ensure they were comprehensive and could be reliably applied to the data. Once coding was complete, coding frequency tables and matrices were developed to identify reoccurring codes (i.e., themes) and to pinpoint experiences that differed between YW and men. Photographs/descriptions were also reviewed for topics that were related to men’s involvement and linked to emergent themes. Analytical memos were written to describe themes, together with illustrative quotes and photographs.

### Ethics

The research was approved by the Maseno University Scientific and Ethics Review Committee in Kisumu, Kenya, and the Duke University Health System Institutional Review Board in the United States. All participants provided their written informed consent to participate in the study and allow their photographs and descriptions to be used for research purposes.

## Results

### Participants

Participants’ photographs were discussed in 12 same-gender group discussions with YW and four same-gender group discussion with men; a total of 22 YW and 17 men participated in one or more of the discussions. Each group discussion included between 6 and 10 participants. Four mixed-gender group discussions were also conducted, and 20 YW and 16 men who participated in the same-gender group discussions also participated in one or more mixed-gender group discussions. Each mixed-gender group discussion included 9–21 participants; participants could participate in up to two mixed-gender group discussions. Eighteen YW participated in an IDI. Participants represented a mix of marital statuses (married or not) and living situations (living with partner or not), although many were married and living with their partner. Most participants had completed primary-level education and were self-employed or a housewife (for women), or engaged in agricultural work, the fishing industry, or other non-formal occupation (Table 1).

**Table 1:** Characteristics of young women (YW) and men in intra-cohort discussions and characteristics of YW in in-depth interviews

	Same-gender group discussions (*N* = 16)		Mixed-gender group discussions (*N* = 4)		In-depth interviews
	YW (*n* = 22)	Men (*n* = 17)	YW (*n* = 20)	Men (*n* = 16)	YW (*n* = 18)
**Age (years)**					
Mean	22.45	28.06	22.45	28.13	22.6
Stand Dev.	1.30	6.19	1.36	6.39	1.29
Range	20–24	21–47	20–24	21–47	20–24
**Marital status**					
Married and living with partner	14	10	14	9	12
Not married and not living with a partner	5	5	3	5	3
Not married and living with partner	1	-	1	-	1
Married and not living with partner	1	2	1	2	1
Widowed	1	-	1	-	1
**Highest level of education**					
Some primary school	8	2	7	2	5
Completed primary school	7	4	7	4	7
Some secondary/high school	2	1	2	1	2
Completed secondary/high school	5	9	4	8	4
Post- secondary certificate	-	1	-	1	-
**Main occupation**					
Not employed (non-student, non-housewife)	3	-	2	-	2
Housewife	5	NA	5	NA	4
Self-employed, market/street vendor	9	4	9	4	8
Boda (commercial motorbike taxi) driver	-	6	-	6	-
Bar, tavern, club, hotel employee	1	-	1	-	1
Agricultural work	1	6	1	5	1
Fishing industry	-	1	-	1	-
Student	1	-	1	-	1
Peer educator	1	-	1	-	1

### Men’s influences on young women’s sexual health

**Fig. 1:**
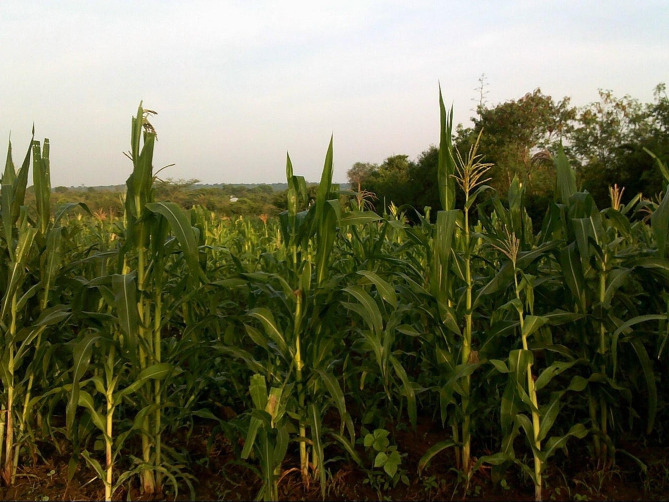
It’s a must we finish customs whether you like it or not. When you harvest or plant, you must perform sexual rituals. It’s a Luo custom. When you harvest, even if you are sick or not, you must do sexual rituals. This violates the rights of women in relation to sex because no one will understand you when she doesn’t want [to fulfill the custom]. (24-year-old young woman)

In describing their photographs, YW in the same-gender group discussions explained that they had little autonomy over their sexual lives and that there are cultural, economic, and social factors that tend to define when and how sexual encounters occur for women. Participants said that YW may be obligated to engage in sex to garner financial support from a male partner due to a lack of personal wealth or control of finances. YW also discussed that women are expected to have sex at their partner’s discretion, even during times when the woman is feeling ill or has little or no interest in having sex. YW described cultural practices where women are obligated to engage in unprotected (condom-less) sex in observance of building a house, to mark seasons of food production, or upon the death of a spouse or parent. A 24-year-old YW’s photograph of a field of maize highlighted the pressure women face to perform expected customs (Fig. 1).

### Men’s influence on young women’s risk for HIV

During the same-gender group discussion of photographs among YW, a restricting male influence on YW’s risk for HIV emerged in the majority of photo assignments. YW described that the behaviours of the men in their lives were their primary risk factor for HIV. Several photographs were singled out as describing situations with men that place YW at heightened risk for HIV. Men’s behaviour, manifested in intimate partner violence, forced sex/rape, instigating couple disharmony then expecting sex (unprotected in most cases) from their wives/girlfriends as a way of resolving differences, and infidelity, were identified as ways men increase YW’s risk for HIV. Several photographs were also presented depicting situations with men that place YW at a reduced risk of acquiring HIV. These situations included using condoms, having monogamous sexual partnerships, or settling in marriage to avoid infidelity.

### Men’s influence on young women’s PrEP use

In the IDIs, YW spoke about their relationships with their husbands in general. They often said that they view their husbands as the primary and most important source of financial and emotional support in their lives. Although most YW acknowledged that they receive helpful advice from sources other than their husbands, including friends and mothers, their husbands were important to consult when making decisions about family-related issues. Several YW reported receiving negative and discouraging comments from husbands at times, including disparaging remarks about her or her family.

**Fig. 2:**
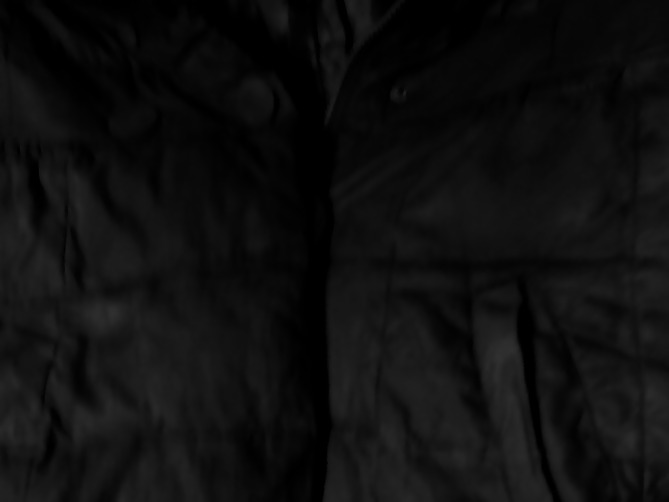
This is a jacket for my husband. He is the one who makes it difficult for [me] to take PrEP. He denies me not to take PrEP. He beats me whenever I take PrEP. He doesn’t know the importance of PrEP. (24-year-old young woman)

**Fig. 3:**
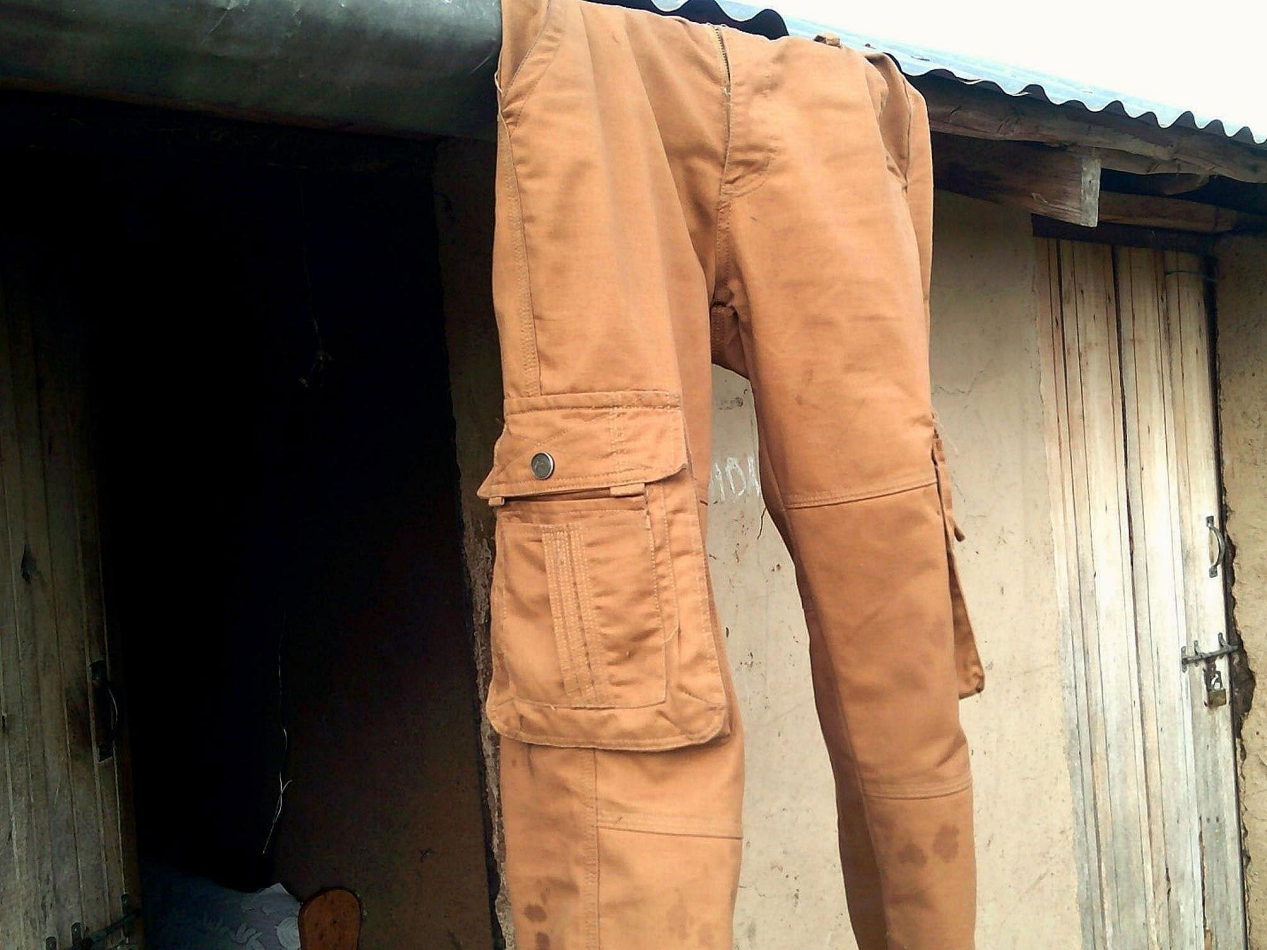
The trouser represent a husband, in this case. It becomes difficult for an adolescent girl and young woman to take PrEP [because] husbands don’t allow their wives (partners) to take PrEP as they believe that someone who takes PrEP is a prostitute, or that some take it as a form of family planning. This is because husbands lack knowledge about PrEP and they need to be taught on benefit of taking PrEP. (22-year-old young woman)

YW in same-gender group discussions emphasized that men are often viewed as barriers and not as supporters of PrEP. YW presented multiple photographs illustrating how men can negatively influence YW’s daily use of PrEP by overtly ordering their female partner to stop taking PrEP or threatening violence (Fig. 2). When describing her photograph (Fig. 3), a 22-year-old YW noted that men are not educated about PrEP and thus have limited knowledge or misinformation about what it is:

*That situation occurs because I can say when we were being taught about PrEP, we were only taught as women alone, we were not taken with our men. So they lack knowledge about PrEP. That’s why it brings difficulty between a man and woman for a woman to find a way of taking PrEP. (22-year-old YW)*

**Fig. 4:**
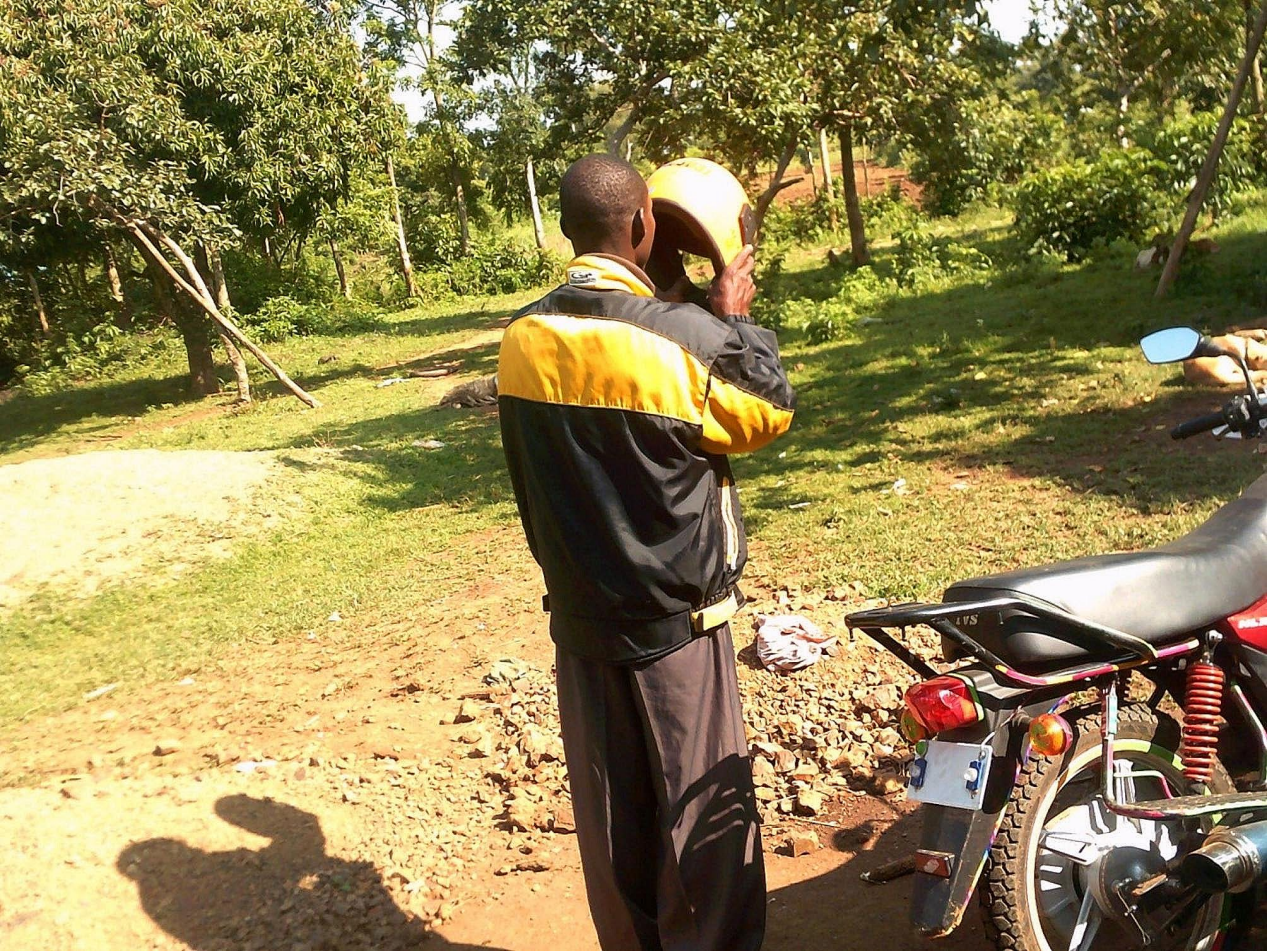
A man putting on a jacket colored black and yellow, and he is standing next to the motorbike carrying the motorbike helmet. He reminds me and he encourages me to use PrEP. (23-year-old young woman)

The YW suggested that greater social support from male family members, partners, and peers by means of reminding YW or encouraging them to take PrEP daily would be beneficial in encouraging daily PrEP adherence and continued use. During their same-gender group discussions, YW described that emotional and instrumental support from men in their lives could lead to increased use of PrEP. A YW described how her husband supported her use of PrEP by encouraging her to take her pills and providing transportation assistance and/or accompanying her for PrEP refills. When describing her photograph (Fig. 4), the participant said:

That is my husband and he always encourages me to take PrEP. Even when I am busy, I can always send him to go and collect for me PrEP from the hospital or at times he can always take me by motorbike when I am going to collect PrEP. He encourages me to use PrEP as he takes care of my life and the life of the children. (23-year-old YW)

On their part, the men in their same-gender group discussions said that the stigmatizing information often heard in the community is to blame for persuading men and women to believe that PrEP is not an appropriate HIV prevention option. They explained that beliefs that PrEP is a contraceptive, can negatively affect foetal development, is an HIV treatment drug, or is only used by sex workers can negatively influence men’s support of YW’s use of PrEP. Men also expressed that other forms of HIV prevention promoted in the community are more commonly accepted, such as condoms and monogamy, which then also negatively affects a man’s support of PrEP and thus a YW’s ability to adhere to PrEP.

Men suggested that male peers and partners of YW could positively influence women’s use of PrEP by way of encouraging and reminding them to take PrEP and also talking more openly about PrEP use among their male and female neighbours.

### Enhancing men’s support of young women’s PrEP use

YW in the group discussions described valuing the idea of having male support of PrEP through emotional and instrumental assistance. Some YW described ways in which their partners and male friends/family actively supported their PrEP use. A woman explained how she gained her partner’s support: drawing upon her own experience of male support (Fig. 4), the 23-year-old YW advised other YW to openly discuss their interest in or use of PrEP with their partner and to reach out to community health volunteers (CHVs) and healthcare workers, if necessary, to talk to their partners about the benefits of PrEP. She encouraged YW to come to an agreement with their partners about how and when PrEP can be used. She said:

*So, [for other] young women, when you want to go and take PrEP or any time that you want to do anything, you should share with your spouse so that he may know that you are using such a thing. You should not hide from him. Use it openly…We can look for CHVs or doctors [healthcare providers] who are available in the community so that they continue to encourage men to encourage their wives on PrEP use. (23-year-old YW)*

Most men in their same-gender group discussions were of the view that male partners and peers can support persistent PrEP use among YW by sharing helpful information about PrEP with men and women in the community, encouraging and reminding their partners to take PrEP, providing money for transport to the clinics, and relieving them of other burdens that may hinder persistent PrEP use. They proposed that men should talk with their partners and come to an agreement about how PrEP should be used in their household. They also proposed that men should demonstrate their support for their partner by taking PrEP themselves. Participants said that, at the very least, men should remain faithful and loving to their partners and protect themselves from being exposed to HIV.

**Fig. 5:**
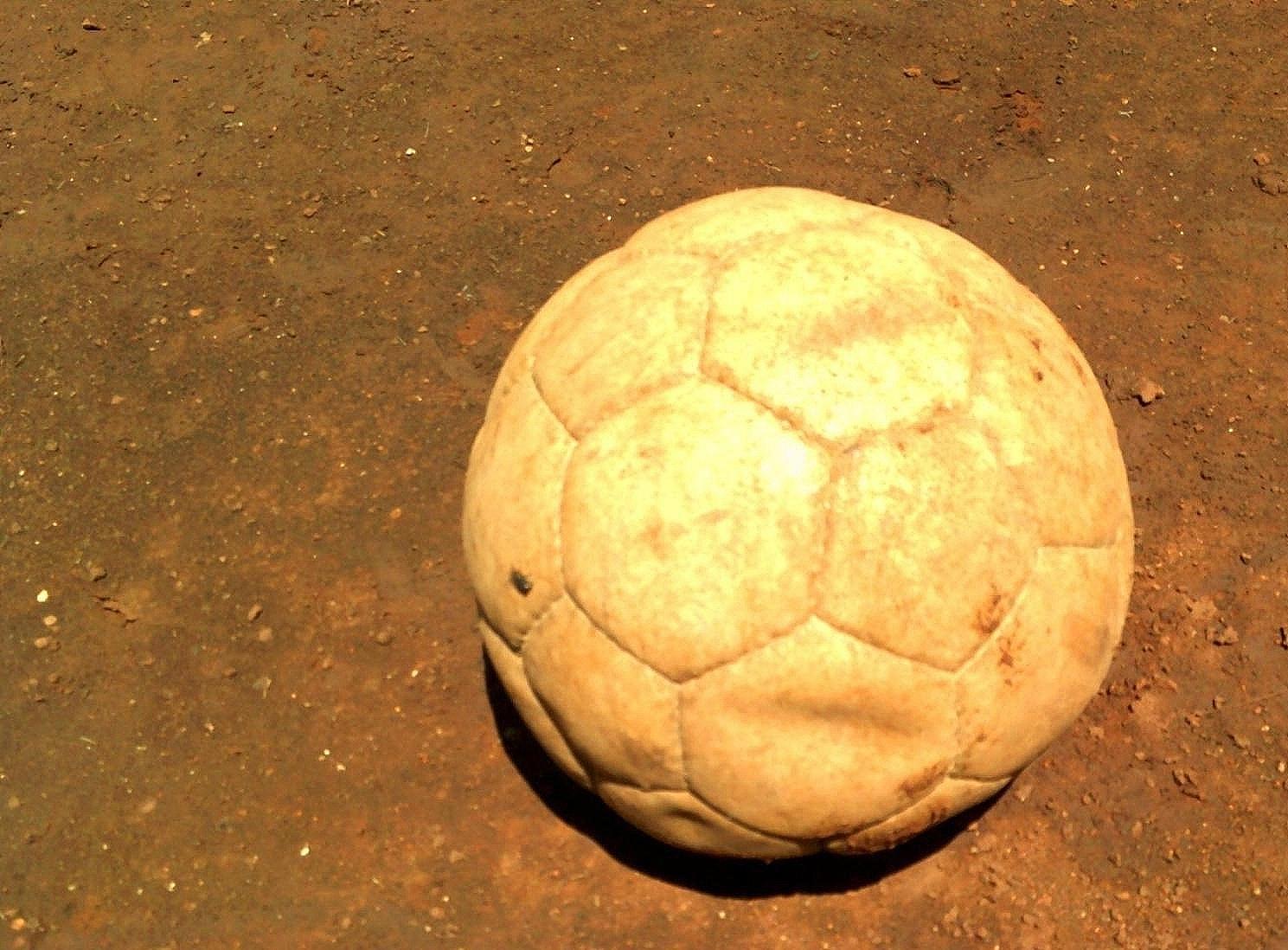
A ball placed on the playing ground. Those who stay in our community use football to associate with young women who join these clubs for practice. When they associate and share ideas there, young women can be sensitized on the importance of using PrEP. (21-year-old male partner of a young woman)

Men also expressed that PrEP use among YW would be supported if its use was better promoted in the community and men were more knowledgeable about PrEP in general. They suggested community-based programs to educate men about PrEP and ideally normalize the use of PrEP as an HIV prevention option. They also supported continuing PrEP education to motivate current PrEP users, hosting group meetings to discuss PrEP with elders, leading education sessions themselves, and providing more reference materials about PrEP in the community. Men suggested promoting the benefits of PrEP on the radio and TV dramas; in schools, churches, and Chiefs’ barazas (camps); and through athletic clubs. While commenting on a photograph of a football symbolizing male athletic clubs (Fig. 5), a 21-year-old male participant said:

We can also organize some tournaments. Then, people will come and there the main agenda – which will be in the tournament – will be how we help our young women to use this PrEP. I think that one will be very fruitful to our young women. (21-year-old male partner)

Similar sentiments were also observed during the mixed-gender group discussions. There was acknowledgment that men share in the responsibility to prevent the spread of HIV in the community, and the participants were supportive of men promoting PrEP use among women. Men described supporting women’s use of PrEP because it was a way to protect the health of women and families:

*I wanted to say that it’s a woman who takes care of the baby most, and a woman can only take care of the baby while she is healthy. So you tell a woman if you are unhealthy, you can’t take care of a baby. But when you take PrEP, you can live longer. Then you find you are encouraging women to take PrEP. (Male in mixed-gender group discussion)*

In addition, boda boda (commercial motorbike taxi) riders, who are often male, were identified as a potential group that could have an important influence on the community views of PrEP. Participants noted that boda boda riders can often spread disparaging rumours or misinformation about PrEP, yet if they were properly sensitized about the positive benefits of YW’s PrEP use, they could be a powerful social influencer:

*These motorbike drivers can be bought [for] or issued PrEP-branded reflectors. So they put them on as they ride across the town, so that many people can see their backs branded “PrEP, PrEP”. They will ask each other, “What is PrEP,” and they go into details to find out what PrEP is. From that juncture, the campaign would be accomplished. (Male in mixed-gender group discussion)*

Participants said they were hopeful that broader male knowledge and acceptance of PrEP would create a more conducive environment for women to use PrEP more freely.

## Discussion

PrEP use by young women at elevated risk of HIV is sub-optimal in most sub-Saharan African countries, including Kenya, due to, in part, lack of support from male partners [49]. Our findings showed that women believe they have limited autonomy over their sex lives due to cultural, economic, and relational factors. By extension, men were also perceived as contributing to YW’s risk for HIV. Women’s autonomy over their sex life is a significant predictor of a number of sexual and reproductive health outcomes [50]. Specifically, gender norms and power dynamics influence men’s acceptance and support of women’s use of HIV prevention products, such as PrEP [48, 51]. Even though PrEP is part of the expanding set of female-controlled and female-initiated strategies in preventing HIV infection among women [52, 53], gender norms and inequalities increase women’s vulnerability to HIV due to multiple factors. These include a limited ability to negotiate safer sex, engagement in transactional sex, and a curtailed ability to test, disclose, and access HIV treatment because of fear of violence or abandonment [1]. The desire to avoid violence and/or preserve their relationship and the trust of their partner may weigh more heavily on women’s lives than HIV prevention considerations [35, 42, 54].

The YW in our study viewed men’s behaviour and attitudes as a barrier to women’s PrEP use. They illustrated how men negatively influence women’s daily use of PrEP by overtly ordering their female partner to stop taking PrEP or threatening violence. In heterosexual relationships, the absence of support and disapproval by male sexual partners is a major barrier to women’s PrEP use [34, 55, 56]. It has also been demonstrated that men have a critical influence on women’s ability and willingness to negotiate and use HIV prevention methods [57] and that relationship power dynamics affect how women make decisions about the use of sexual and reproductive health products [58]. In their study in Kenya, Haberer et al. [39] found that YW can best take PrEP when their male partners encourage them to do so.

Men in this study felt that male peers and partners of YW can positively influence YW’s use of PrEP by way of encouraging and reminding them to take PrEP and also talking more openly about PrEP use in their households and in the community. Male participants also stressed that increased PrEP awareness and knowledge among male peers and male partners is key to enhancing their support of YW’s PrEP use. However, male partners’ support of YW’s PrEP use is often dependent on the condition that women disclose their decision to use PrEP prior to actual use and engage their male partner in the decision-making process [49, 58]. Disclosure has been described as an empowering way to address PrEP non-adherence [59, 60].

Our findings suggest that disclosure of a woman’s PrEP use to the men in their lives may lead to increased use of PrEP. This is in line with other research findings on women’s PrEP use and partner disclosure. In a study that explored the factors that influenced PrEP adherence among women in Harare, Zimbabwe and Cape Town and Johannesburg, South Africa from 2016 to 2018, participants shared that they proactively discussed PrEP in their communities, which improved their ability to take PrEP and encouraged others to use PrEP [61]. Between 2016 and 2018, the EMPOWER trial explored the dimensions of PrEP use stigma among AGYW in sub-Saharan Africa and also showed that disclosure of PrEP use to a male partner was associated with less reported stigma and hence greater PrEP adherence [62].

In the group discussions, the men felt that stigmatizing information about PrEP or the type of people who take PrEP shared in the community hinders YW’s PrEP use. In other studies, women have also cited stigma and negative narratives about PrEP in the community as barriers to PrEP uptake and sustained use [38, 55, 56, 63]. The anticipated stigma of being perceived as a person living with HIV as a result of PrEP use can be a deterrent for both women and their male partners, with women fearing the additional stigma of being considered sexually promiscuous [38, 64]. The negative community narratives on PrEP use among YW were echoed by the men. The male participants suggested that PrEP stigma can be addressed by normalizing PrEP use through community awareness, offering continued PrEP education to motivate users, hosting group meetings to discuss PrEP with elders, and providing more reference materials about PrEP in the community. Jani et al. [38] recommend couples counselling to educate men and women on the benefits of PrEP, which can increase PrEP acceptance and adherence among women. The men in our study also shared that men may be more interested in supporting YW’s PrEP use if PrEP was promoted as a means of protecting the health of women and families.

The potential limitations of our study include those common to participatory research such that if different participants were enrolled, different findings may have been identified than those presented here. Some participants may have also shared socio-desirable responses or felt they could not fully share the context about their photographs in front of others, although we took several steps to limit these outcomes from occurring.

In moving forward, men should be included in PrEP community awareness initiatives that focus on YW, to educate them on the benefits of PrEP within their relationship and to help them to address their own concerns about HIV and sexual and reproductive health. To reduce PrEP stigma, there is a need to address the broader community, which requires investing in appropriate PrEP community awareness initiatives that normalize PrEP use within a community-wide HIV prevention framework. This has been proven to work in PrEP programs, such as DREAMS, which foster community and clinic-based discussions and PrEP adherence clubs, thereby normalizing PrEP use [38].

## Conclusions

YW perceive they have limited autonomy over their sex lives due to cultural, economic, and relationship dynamics. Men have been perceived as contributing to women’s risk of HIV infection, yet they remain a barrier to women’s use of PrEP. Stigmatizing information in the community likely persuades men and women to believe that PrEP is not an appropriate HIV prevention option. Male partners and peers can positively influence women’s use of PrEP by encouraging and reminding them to take PrEP and talking more openly about PrEP in the community. Men can better support women’s PrEP use when PrEP use is normalized through community awareness.

## Data Availability

The data that support the findings of this study are available from the corresponding author upon reasonable request.

## Abbreviations

AGYW: Adolescent Girls and Young Women
CHV: Community Health Volunteers
DREAMS: Determined, Resilient, Empowered, AIDS-free, Mentored, Safe
FEM-PrEP: Female Pre-Exposure Prophylaxis
HIV: Human Immuno-deficiency Virus
IDI: In-depth Interview
PrEP: Pre-Exposure Prophylaxis
WHO: World Health Organization
YW: Young Women
